# Single-Molecule Telomere Assay via Optical Mapping (SMTA-OM) Can Potentially Define the ALT Positivity of Cancer

**DOI:** 10.3390/genes14061278

**Published:** 2023-06-16

**Authors:** Kaitlin Raseley, Zeal Jinwala, Dong Zhang, Ming Xiao

**Affiliations:** 1 School of Biomedical Engineering, Drexel University, Philadelphia, PA 19104, USA; ker343@drexel.edu (K.R.); jinwala.zeal@gmail.com (Z.J.); 2 Department of Biomedical Sciences, College of Osteopathic Medicine, New York Institute of Technology, Old Westbury, NY 11568, USA; 3 Center for Cancer Research, New York Institute of Technology, Old Westbury, NY 11568, USA; 4 Center for Genomic Sciences and Center for Advanced Microbial Processing, Institute of Molecular Medicine and Infectious Disease, Drexel University College of Medicine, Philadelphia, PA 19102, USA

**Keywords:** alternative lengthening of telomeres (ALT), genomics, cancer telomeres, U2OS, SK-MEL-2, Saos-2, UMUC3, LNCaP, IMR90 senescence, single molecule optical mapping

## Abstract

Telomeres play an essential role in protecting the ends of linear chromosomes and maintaining the integrity of the human genome. One of the key hallmarks of cancers is their replicative immortality. As many as 85–90% of cancers activate the expression of telomerase (TEL+) as the telomere maintenance mechanism (TMM), and 10–15% of cancers utilize the homology-dependent repair (HDR)-based Alternative Lengthening of Telomere (ALT+) pathway. Here, we performed statistical analysis of our previously reported telomere profiling results from Single Molecule Telomere Assay via Optical Mapping (SMTA-OM), which is capable of quantifying individual telomeres from single molecules across all chromosomes. By comparing the telomeric features from SMTA-OM in TEL+ and ALT+ cancer cells, we demonstrated that ALT+ cancer cells display certain unique telomeric profiles, including increased fusions/internal telomere-like sequence (ITS+), fusions/internal telomere-like sequence loss (ITS−), telomere-free ends (TFE), super-long telomeres, and telomere length heterogeneity, compared to TEL+ cancer cells. Therefore, we propose that ALT+ cancer cells can be differentiated from TEL+ cancer cells using the SMTA-OM readouts as biomarkers. In addition, we observed variations in SMTA-OM readouts between different ALT+ cell lines that may potentially be used as biomarkers for discerning subtypes of ALT+ cancer and monitoring the response to cancer therapy.

## 1. Introduction

Human telomeres consist of unique repetitive DNA sequences, (TTAGGG)n, which are found at the terminal ends of each chromosome and function as protective barriers from the attrition of protein-coding regions [1,2]. In normal somatic cells, telomere length is approximately 10–15 kilobases (kb) [3,4,5], but over time, telomeres will inevitably shorten with each cell division in the absence of a telomere maintenance mechanism (TMM). In the case of neoplastic cells experiencing rampant proliferation, the vast majority of them initiate two TMMs, namely, the re-activation of telomerase (TEL+) and the adoption of the Alternative of Lengthening of Telomeres (ALT+) pathway, to avoid excessive shortening of telomeres, which could lead to cell cycle arrest, or senescence, or cell death. Approximately 10–15% of cancers lack detectable telomerase activity but are still capable of maintaining telomere integrity using the ALT pathway [6,7]. Unlike human telomerase, which directly adds more telomere tract sequences to the pre-existing telomeres, the ALT+ cells rely on homology-dependent repair (HDR) to maintain their telomeres, which may result in unique structural changes at the telomeres and subtelomeres.

The ALT pathway is more prevalent in certain types of cancer, including neuroblastoma (NB), pancreatic neuroendocrine tumors (PanNET), osteosarcoma, and glioma [8,9]. For some, such as the PanNET, the ALT positivity predicts a likely worse prognosis. However, for others, such as NBs, the opposite is often true. Mechanistically, DNA damage response (DDR) and HDR proteins play an essential role in the ALT pathway [7]. Recent studies from multiple groups have shown that a unique HDR pathway, the Break-Induced Replication (BIR) pathway, is critical for the ALT [10,11,12,13,14]. The potential templates for the BIR include the telomere from the homologous chromosome, from a nonhomologous chromosome, or even from the extrachromosomal telomeric repeats (ECTRs), which are abundant in the ALT+ cells [7,15]. Identification of the TEL+ cells primarily relies on the well-established telomerase repeated amplification protocol (TRAP) assay [16]. However, the identification of the ALT+ cells is much more tedious and has to rely on the positivity of multiple assays, including telomere fluorescent in situ hybridization (Telo-FISH), ALT-associated acute promyelocytic leukemia bodies (APBs), telomere dysfunction induced foci (TIFs), telomere sister chromatin exchange (tSCE), C-circle, and others [7,9]. Most recently, using machine learning and whole genome sequencing (WGS), Lee and colleagues proposed that telomere variants may potentially be used to differentiate ALT+ cells from TEL+ cells because they are generated from distinct mechanisms [17,18]. Therefore, novel and more reliable biomarkers/assays are urgently needed for ALT research as well as for identifying the ALT+ tumors in the clinic for targeted therapy in the near future.

Irrespective of ALT+ or TEL+ cells, detecting and quantifying the genome-wide changes of telomeres at individual chromosome arms have been challenging [19]. Current assays that have been used for this purpose include Q-PCR (quantitative polymerase chain reactions), Q-FISH (quantitative fluorescent in situ hybridizations), STELA (single telomere length analysis), and TeSLA (telomere shortest length assay) [19]. As discussed in detail by Lai and colleagues, every one of these approaches has its limitations and is not easily applicable to genome-wide characterization of telomeres for both TEL+ and ALT+ cells.

We recently developed a novel method that enables genome-wide analysis of telomeres at the single-molecule level, called Single-Molecule Telomere Analysis via Optical Mapping (SMTA-OM) technology [20]. As whole-genome optical mapping can identify each subtelomeric region adjacent to the telomeres [21,22], SMTA-OM assay can identify and characterize each telomere and its associated features. Using the SMTA-OM, we analyzed the telomere/chromosome end status of two TEL+ cancer cell lines (LNCaP and UMUC3) as well as a senescent primary lung fibroblast cell line, IMR90-S. Subsequently, we utilized the SMTA-OM to characterize the telomere/chromosome end status of three ALT+ cell lines (Saos-2, SK-MEL-2, and U2OS) [23]. From these two recent studies, we demonstrated that the SMTA-OM can be used to visualize and quantify telomeres at the single-molecule level of a specific chromosome arm and with a wide range in length, from 100 base pairs (bp) to over 100 kilobases (kb) [20]. Other telomere/chromosome end features that can be detected and quantified by the SMTA-OM include ECTRs and telomere-free ends (TFEs). Most intriguingly, the SMTA-OM is particularly effective in detecting and quantifying the telomere/chromosome end fusion events in the ALT+ cells. The detected fusions include the fused molecules with internal telomere-like sequences (fusion/ITS+) and fused molecules with no detectable internal telomere-like sequences (fusion/ITS−).

Here, we further analyzed the SMTA-OM results in both ALT+ and TEL+ cancer cells as well as the IMR90-S cells and demonstrated that the SMTA-OM alone can potentially be used to define the ALT positivity through the following readouts/parameters: (1) the presence and abundance of fusion/ITS+; (2) the presence and abundance of the fusion/ITS−; (3) the presence and abundance of TFEs; (4) the presence and abundance of super-long telomeres; and (5) the heterogeneity of telomere lengths at the whole-genome level. We propose that these SMTA-OM readouts can potentially be used to identify the ALT positivity in tumors in the clinic for targeted therapy.

## 2. Materials and Methods

### 2.1. Cell Preparation and High Molecular Weight DNA Extraction

The three ALT+ cell lines, U2OS, Saos-2, and SK-MEL-2, were purchased from American Type Culture Collection (ATCC). Both U2OS and Saos-2 were cultured in McCoy’s 5a medium supplemented with 10% and 15% fetal bovine serum (FBS), respectively. SK-MEL-2 was cultured in Eagle’s Minimum Essential Medium supplemented with 10% FBS. UMUC3 was cultured in Eagle’s Minimum Essential Medium containing Earle’s salts, nonessential amino acids (NEAA), and L-glutamine (2 mM) with 10% FBS. LNCaP cells were purchased from ATCC and cultured in RPMI 1640 with 2 mM L-glutamine, 10 mM HEPES, 1 mM sodium pyruvate, 4500 mg/L glucose, and 1500 mg/L sodium bicarbonate and 10% FBS. IMR90-S cells were purchased from Coriell Cell Repository and cultured in EMEM with Earle’s salts, NEAA, and 2 mM L-glutamine supplemented with 15% FBS. Cells were passaged using 0.25% trypsin-EDTA, except for IMR90-S, which was passaged with 0.05% Trypsin-EDTA. High molecular weight DNA extraction, guide RNA (gRNA) preparation, and the two-color DNA labeling process were conducted as described previously in McCaffrey et al. [20].

### 2.2. Loading and Imaging of DNA

The DNA samples were treated with Protease and IrysPrep Stop Solution (BioNano Genomics, San Diego, CA, USA) [20]. The labeled DNA was stained with YOYO-1 (Invitrogen, Carlsbad, CA, USA) before being loaded into the nanochannels following a previously established procedure [24]. A total of 180 Gb data, which is about 60× coverage, provided around 30 molecules with telomeres for each chromosome arm. The image analysis was performed following the established method [20].

### 2.3. Telomere Length Analysis

Telomeres were identified as the extra labels at the end of a DNA molecule or in the middle of a fused DNA molecule. The fluorescence intensity was used to infer the length of each individual telomere as described in the previously established protocol [20].

## 3. Results

**Figure 1:**
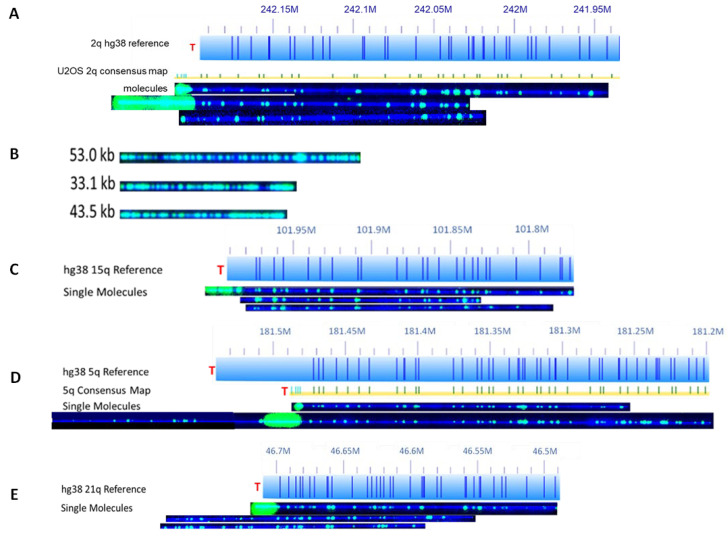
A variety of telomere and chromosome end features that are detected by the SMTA-OM [23]. (**A**) Chromosome ends with highly variable sizes of telomeres of the chromosome arm 2q. (**B**) Extrachromosomal telomeric repeats (ECTRs). (**C**) Chromosome ends without detectable telomere signals, or telomere-free ends (TFEs) of the chromosome arm 15q. (**D**) A fusion molecule with internal telomere-like sequence (fusion/ITS+) derived from the chromosome arm 5q. (**E**) Two fusion molecules without internal telomere-like sequence (fusion/ITS−) of the chromosome arm 21q. “T” in red font indicates the telomere location of a specific hg38 chromosome arm. The discrete and less intense green labels on a DNA molecule were used to align with the consensus map and assist in assigning the DNA molecule to a specific chromosome arm.

In SMTA-OM, first developed by McCaffrey et al., high-molecular-weight genomic DNA (gDNA) molecules were first nicked by a nickase, Nt. BspQI, at recognition sequence 5′-GCTCTTC-3′ [20]. The nick sites across the whole genome were then tagged with a green fluorophore by Taq DNA polymerase. An in vitro CRISPR/Cas9 sgRNA-directed nickase system then directed the specific labeling of telomeric DNA, with the same-colored fluorophore also by Taq DNA polymerase. All DNA molecules were also stained with YOYO-1 (blue). The two-colors-labeled long chromosomal fibers (>150 kilobases (kb)) were linearized in the NanoChannel Arrays (purchased from Bionano Genomics) and imaged. Figure 1 shows the representative images of the two-colors-labeled telomeres/chromosome ends and ECTRs from U2OS cells [23].

**Figure 2:**
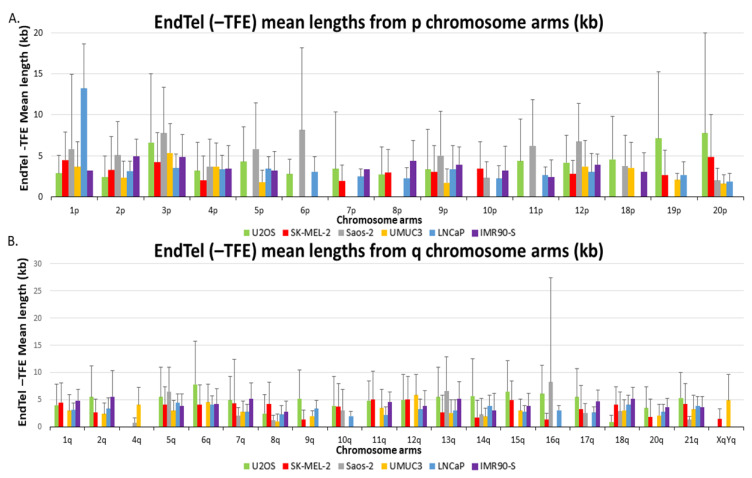
Analysis of the end telomere (-TFE) mean lengths and standard deviations from U2OS (green), SK-MEL-2 (red), Saos-2 (gray), UMUC3 (yellow), LNCaP (blue), and IMR90-S (purple). (**A**) End telomere (-TFE) mean lengths from all the p chromosome arms that were detected by the SMTA-OM. (**B**) End telomere (-TFE) mean lengths from all q chromosome arms that were detected by the SMTA-OM. Chromosome arms that are not depicted did not have measurements from at least one ALT+ and one TEL+ or senescent cell line for comparison.

The pattern of the green color from the Nt. BspQI nick labeling, which only labels the non-telomeric DNA, is used to match a particular chromosome arm based on the human hg38 reference genome. The intensity of the telomere labeling, found at the end of a particular chromosome arm, is proportional to the length of the telomere, and thus is used to measure the length of the telomere. When optimized, the SMTA-OM can detect a telomere with as few as 100 bp and as high as over 100 kb. SMTA-OM can identify the following telomeric/chromosome end features in both ALT+ and TEL+ cancer cell lines (Figure 1): (1) Visualizing and quantifying the heterogeneity of telomeres at the single-molecule level of a specific chromosome arm (Figure 1A); (2) Detecting super-long telomeres. For example, one telomere of chromosome arm 16q of Saos-2 measures at 62.8 kb. In stark contrast, the longest telomere measured from one of the TEL+ cancer cells was from LNCaP 1p at 23.5 kb long; (3) Detecting ETCRs, which can consist of up to 40% of the total telomere sequences in ALT+ cells (Figure 1B). The average length of ECTRs in U2OS is 11 kb, with the longest one measuring at 53 kb (Figure 1B); (4) Detecting and quantifying the telomere-free ends (TFEs) (Figure 1C); (5) Detecting and quantifying the fused molecules with internal telomere-like sequence (fusion/ITS+, or ITS+), in which they have an additional fragment from unknown origins fused to the telomere of a molecule belonging to a specific chromosome arm (Figure 1D); (6) Detecting and quantifying the fused molecules without internal telomere-like sequence (fusion/ITS−, or ITS−) (Figure 1E). Occasionally, the fused fragments share the same green color labeling pattern (such as two molecules from 21q in Figure 1E), indicating that they likely originated from the same two chromosomes.

### 3.1. Summary of Telomere/Chromosome End Characterization Using SMTA-OM

Out of 46 chromosome arms, we were able to measure molecules from 33 arms of SK-MEL-2, 28 arms of Saos-2, 34 arms of U2OS, 34 arms of LNCaP, 28 arms of UMUC3, and 27 arms of IMR90-S. Chromosome arms that could not be measured were due to a lack of either reference or assembled consensus contigs covering our regions of interest. Raw measurements were collected as outlined by McCaffrey et al. [20].

Mean telomere lengths, standard deviations, and percentages of molecules with TFE, ITS+, and ITS− for each analyzed chromosome arm were calculated for each cell line (Supplementary Table S1). The overall count column includes the total number of molecules analyzed for a specific arm. EndTel counts (-TFE) includes the total number of molecules with end telomeres while excluding those with TFEs. TFE count is the total number of molecules without any detectable telomere signal, but the labeling pattern aligns with the reference in the subtelomere region. ITS (the sum of ITS+ and ITS−), ITS+, and ITS− counts are determined in the same manner as mentioned above for the other parameters.

Next, to evaluate the mean telomere length of each arm from the end telomeres, we summed measurements from a chromosome arm and divided them by the corresponding count (Supplementary Table S1). The same method was used to calculate the average lengths of ITS+ molecules. The longest measured telomere for each arm is also listed (column E, labeled as Overall Max).

We were also interested in the frequency of TFE, ITS+, and ITS− occurrence in each chromosome arm per cell line (Supplementary Table S1, columns N, T, V). The percentage for EndTel (-TFE) was calculated by dividing the number of molecules with end telomeres by the total number of molecules for an individual chromosome arm and multiplying by 100. TFE%, ITS%, ITS+%, and ITS−% were all calculated in a similar manner.

All the mentioned calculations were completed for the IMR90-S cells, the three ALT+ cell lines and the two TEL+ cell lines.

### 3.2. ALT+ Cells Manifest Elevated Fusion/ITS+ and Fusion/ITS−

ALT+ cells can be recognized by their unique characteristics of the telomeric/chromosome end regions, such as the elevation of fusion/ITS+ and fusion/ITS−, which are the unique characteristics of ALT+ cells (U2OS, SK-MEL-2, and Saos-2) compared to TEL+ cells (UMUC3, LNCaP) and IMR90-S. Approximately 9% of SK-MEL-2, 19% of Saos-2, and 35% of U2OS molecules are fusions (Table 1). Strikingly, LNCaP, UMUC3, and senescent IMR90 are completely absent of the fusions. On average, ITS+ was observed in 8.6% of all SK-MEL-2, 18% of all Saos-2, and 23% of all U2OS molecules. Although the amount of fusion/ITS− in ALT+ cancer cells is relatively lower, it is still elevated in all three ALT+ cancer cells. Fusion/ITS− was recorded in roughly 0.5% of all SK-MEL-2, 0.5% of all Saos-2, and 12% of all U2OS molecules (Table 1).

We then calculate the fusion/ITS+ and fusion/ITS− percentage of each individual arm of ALT+ cells (Supplemental Table S1, columns T, V). Fusion/ITS+ and fusion/ITS− percentages vary greatly among individual arms of the three ALT+ cells. For example, 50% of 3q molecules from SK-MEL-2, 87.5% of 3q molecules from Saos-2, and 96.2% of 19q from U2OS are fusion/ITS+, while some chromosome arms, such as 3q from U2OS and 2p from both SK-MEL-2 and Saos-2, have no fusion/ITS+ molecules. For fusion/ITS−, the chromosome arms with the greatest elevation of fusion/ITS− from each ALT+ cell were 1p from Saos-2 (8.6%), 3q from SK-MEL-2 (50%), and 3q from U2OS (69.6%). Most of the chromosome arms from the three ALT+ cells are without fusion/ITS−. Overall, chromosome arm 3q is the most unique chromosome arm with respect to the formation of fusion. For SK-MEL-2 cells, all the molecules containing chromosome arm 3q are fusions (50%—fusions/ITS+; 50%—fusion/ITS−). For Saos-2 cells, the chromosome arm 3q has the most fusions and all of them are fusion/ITS+ (87.5%). For U2OS cells, all the fusions containing chromosome arm 3q are fusion/ITS− (69.6%), which rank it the highest in fusion/ITS− and the sixth highest in total number of fusions. At present, the reason why the chromosome arm 3q is so prone to form fusions is unknown.

Within the three ALT+ cell lines, the percentages of fusion/ITS+ of each chromosome arm (Supplementary Table S1, column T) were used to perform a two-tailed t-test to evaluate whether the fusion/ITS+ percentage is significantly different between the ALT+ cell lines (Supplemental Table S2). For fusion/ITS+, we found U2OS and SK-MEL-2, as well as Saos-2 and SK-MEL-2, to be significantly different from each other, with *p* values of 0.005 and 0.019, respectively (Supplementary Table S2, C27, C29). There was no statistical difference between fusion/ITS+ percentages of Saos-2 and U2OS (*p* = 0.748). The same procedure was followed for fusions/ITS− (Supplementary Table S1, column V). U2OS has a significantly different frequency of fusion/ITS− compared to both SK-MEL-2 and Saos-2 (*p* = 0.003, 0.000, respectively) (Supplementary Table S2, B32 and B33). In contrast to fusion/ITS+, Saos-2 and U2OS have significantly different percentages of fusion/ITS− (*p* = 0.000); however, there was no significant difference in fusion/ITS− when comparing Saos-2 and SK-MEL-2 (*p*-value = 0.239) (Supplementary Table S2, B33, C33, respectively).

Taken together, our data suggest that the fusions (both fusion/ITS+ and fusion/ITS−) not only can be used to distinguish ALT+ cells from TEL+ cells; they could also be useful for differentiating the subgroups of ALT+ cancers.

### 3.3. ALT+ Cells Show Elevated Telomere-Free Ends

The elevation of TFEs in ALT+ cells is another pronounced feature compared to TEL+ cells and senescent IMR90 (Table 1 and Supplemental Table S1, Columns M and N). SK-MEL-2 has the greatest frequency of TFEs at 7.6%. This was followed by U2OS and Saos-2 at 6.4% and 6.3% of molecules with TFEs, respectively. In stark contrast, neither LNCaP, nor UMUC3, nor senescent IMR90 cells had any detectable TFEs. Intriguingly, we observed that certain chromosome arms in ALT+ cells are more likely to experience complete telomere loss than others. The chromosome arms in the SK-MEL-2 with the highest elevation of TFEs were 11q (40%), 6q (24%), and 9q (20%). The arms 19q (85.7%), 1q (42.9%), and 6q (28.6%) had the greatest TFEs from Saos-2. The top three arms with TFEs for U2OS were 14q (50.0%), 3q (30.4%), and 12q (12.5%). Both SK-MEL-2 and Saos-2 have a high incidence of TFEs from chromosome arm 6q. Only one chromosome arm, 5q, was absent of any TFEs from U2OS, SK-MEL-2, and Saos-2. The elevation of TFEs is a characteristic unique to ALT+ cancer cells, since no TFE was recorded for the two TEL+ cells (UMUC3 and LNCaP) and the senescent IMR90. These data indicate that there is heightened spontaneous DNA damage in the subtelomeric regions in all the ALT+ cells, resulting in the complete loss of telomeres.

Within the three ALT+ cells, the percentages of TFEs from each chromosome arm were used to perform a two-tailed *t*-test to evaluate whether the TFE percentage is significantly different between the ALT+ cell lines (Supplementary Table S2). The *t*-test reveals no statistical difference in TFE percentage between U2OS, SK-MEL-2, and Saos-2 cell lines.

### 3.4. Overall Telomere Mean Length and End Telomere (-TFEs) Mean Length Manifest Either Minimal or No Differences between the ALT+ Cells and TEL+ Cells

#### 3.4.1. Overall Telomere Mean Length

The overall telomere mean lengths include measurements from end telomeres, TFEs, ITS+, and ITS−. As shown in Table 1, the overall telomere mean lengths for ALT+ samples were 3.8 ± 5.2 kb for Saos-2, 3.4 ± 5.1 kb for U2OS, and 3.2 ± 3.8 kb for SK-MEL-2. TEL+ cells had overall telomere mean lengths of 3.2 ± 2.2 kb for LNCaP and 3.1 ± 2.8 kb for UMUC3. The overall telomere mean length for IMR90-S is 4.0 ± 2.6 kb. Using the overall telomere mean lengths from all six cell lines, a two-tailed unequal variance *t*-test was performed to evaluate the statistical significance between ALT+ and TEL+ cell lines. Intriguingly, considering most overall telomere mean lengths are within 1 kb of each other across the cell lines, there is little to no difference seen among the ALT+, TEL+, and IMR90-S cells, apart from SK-MEL-2 and IMR90-S at *p* = 0.002 (Supplementary Table S2, B5). Therefore, we conclude that the overall telomere mean length is not a good indicator for differentiating ALT+ cells from TEL+ cells. Overall telomere mean lengths include a mixture of molecules with TFEs and ITS−, which were assigned a length value of 0 kb, and thus will skew the average telomere length.

Consistent with previous findings [25,26], we observed a wide distribution of telomere lengths for ALT+ cells and high variability across individual chromosome arms (Supplementary Table S1), suggesting that, indeed, the lengths of telomeres in ALT+ cells are more heterogeneous than those in the TEL+ cells. Chromosome arm 20p is the only arm where U2OS (7.0 ± 11.5 kb), SK-MEL-2 (4.5 ± 4.7 kb), and Saos-2 (1.9 ± 1.5 kb) all contain telomeres with a longer average length than that of UMUC3 (1.6 ± 1.1 kb) and slightly longer than LNCaP (1.9 ± 1.0). The 20p arm from senescent IMR90 cells was not analyzed, so it is not certain whether the overall telomere mean length would also be less than that of the ALT+ cells. There are no chromosome arms where the overall telomere mean lengths of UMUC3, LNCaP, and senescent IMR90 cells were all longer than U2OS, SK-MEL-2, and Saos-2 cells overall telomere mean lengths.

#### 3.4.2. End Telomere (-TFEs) Mean Length

As mentioned above, the overall telomere mean length of ALT+ cancer cells is skewed toward shorter lengths due to the presence of many TFE and ITS− molecules. The end telomere (-TFEs) mean length, which excludes the TFE and ITS− molecules, may be a better differentiating parameter. As shown in Table 1, the end telomere mean lengths (excluding TFEs and ITS−) for the three ALT+ cells were 5.0 ± 2.2 kb for U2OS, 4.5 ± 5.6 kb for Saos-2, and 3.5 ± 3.9 kb for SK-MEL-2. TEL+ cells and IMR90-S retained the same measurements of 3.2 ± 2.2 kb for LNCaP, 3.1 ± 2.8 kb for UMUC3, and 4.0 ± 2.6 kb for senescent IMR90, since no TFEs or ITS is recorded in these cells. Using all individual end telomere (-TFEs) mean lengths, a two-tailed t-test was conducted to evaluate the significance of the end telomere mean length excluding TFEs and ITS− between ALT+ and TEL+ cell lines (Supplementary Table S2). We found that U2OS is statistically different from both UMUC3 and LNCaP, with *p* values of 0.003 and 0.040, respectively (Supplementary Table S2, C8, D8). However, U2OS is not significantly different from senescent IMR90 (*p* = 0.072) (Supplementary Table S2, B8). The only ALT+ cell line found to be significantly different from senescent IMR90 is SK-MEL-2 (*p* = 0.018) (Supplementary Table S2, B9). No other combinations of ALT+ and TEL+ cells resulted in significant *p* values (Supplementary Table S2). Among the three ALT+ cells studied, only U2OS and SK-MEL-2 significantly varied in end telomere (-TFEs) mean length (*p* = 0.009) (Supplementary Table S2, B44). Therefore, our data indicate that end telomere mean length excluding TFEs and ITS− is not a distinctive feature of ALT+ cells either.

End telomere (-TFE) mean lengths are also visualized in two separate bar graphs representing p and q chromosome arms in Figure 2. The chromosome arms depicted have at least 1 ALT+ cell line and 1 TEL+ cell line for comparison. Certain chromosome arms in ALT+ cells had substantially longer telomeres compared to the corresponding chromosome arm from the TEL+ samples. Similar to the overall telomere mean lengths, 20p is the only arm where all three ALT+ cell lines have end telomere (-TFE) mean lengths greater than the two TEL+ cells and the senescent IMR90, but there are no arms where the TEL+ cells have greater end telomere (-TFE) mean lengths than all three ALT+ cell lines. Taken together, our data indicate that the end telomere (-TFE) mean length for the individual arm is not a useful readout to differentiate ALT+ cells from TEL+ cells either.

### 3.5. ALT+ Cells Have More Super-Long Telomeres

**Figure 3:**
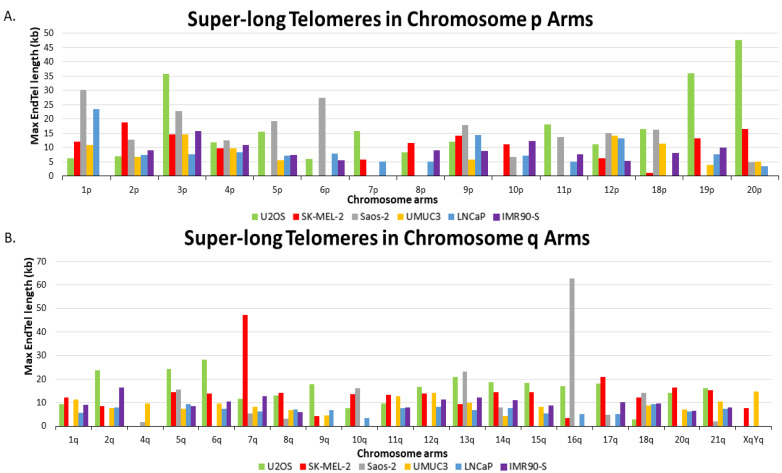
Analysis of super-long telomeres in U2OS (green), SK-MEL-2 (red), Saos-2 (gray), UMUC3 (yellow), LNCaP (blue), and IMR90-S (purple). For all cell lines, the colored bars represent the longest measured telomere from a specific chromosome arm. (**A**) Super-long telomeres from all p chromosome arms that were detected by the SMTA-OM. (**B**) Super-long telomeres from all q chromosome arms that were detected by the SMTA-OM. Chromosome arms that are not depicted did not have measurements from at least one ALT+ and one TEL+ or senescent cell line for comparison.

Interestingly, we observed that the ALT+ cells have more super-long telomeres. As seen in Figure 3, we compiled the longest telomere of each chromosome arm of all six cells and plotted them in a bar graph. Super-long telomeres from p and q chromosome arms in all six cell lines are represented by separate bar graphs. A molecule of 16q from Saos-2 has the longest telomere length of 62.8 kb, while in U2OS and SK-MEL-2, the longest telomeres are molecules from 20p of U2OS with 47.5 kb and 7q from SK-MEL-2 at 47.3 kb, respectively. The longest telomeres of each chromosome arm in ALT+ cells are generally from end telomeres, not from fusion/ITS+. For comparison, the longest telomere from UMUC3 is 14.75 kb from XqYq, the longest telomere from LNCaP is 23.5 kb from 1p, and the longest telomere from senescent IMR90 is 16.4 from 2q.

Out of 38 total chromosome arms analyzed, U2OS has the most chromosome arms (14) with the longest telomeres. These 14 arms are 2q, 3p, 5q, 6q, 7p, 9q, 11p, 12q, 14q, 15q, 18p, 19p, 20p, and 21q. SK-MEL-2 had 10 chromosome arms with the longest telomeres, including 1q, 2p, 7q, 8p, 8q, 10p, 11q, 17q, 19q, and 20q. The same as SK-MEL-2, Saos-2 has the longest telomere for 10 chromosome arms also, which includes 1p, 4p, 5p, 6p, 9p, 10q, 12p, 13q, 16q, and 18q. Note that chromosome arms 3q, 4q, 16p, and XqYq either lack a sufficient number of measurements for analysis or have very few end telomeres in all the ALT+ cells.

A two-tailed t-test using the longest telomeres of each chromosome arm among five cell lines clearly shows that ALT+ cells have statistically more super-long telomeres than TEL+ cells (Supplementary Table S2). Overall, we observe many more super-long telomeres in ALT+ cells. Telomeres longer than 15 kb were recorded for 4.3% of Saos-2, 4.1% of U2OS, and 1.0% of SK-MEL-2. In contrast, 0.4% of LNCaP telomeres and 0.3% of IMR90 senescence telomeres are longer than 15 kb, while UMUC3 had no telomeres measuring longer than 15 kb. Therefore, our data indicate that the presence of super-long telomeres may also serve as an indicator for differentiating ALT+ from TEL+ cells.

### 3.6. ALT+ Cells Show Higher Telomere Length Heterogeneity

**Figure 4:**
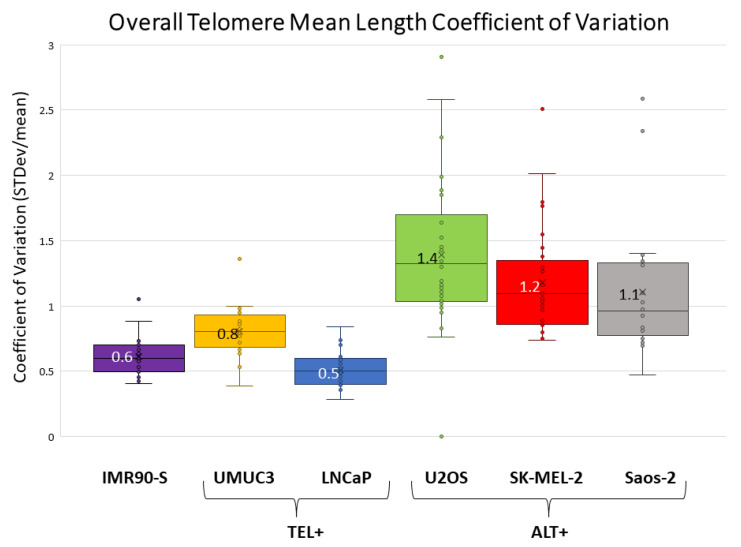
Coefficient of variation (CV) for overall telomere length including end telomeres, TFEs, and ITS measurements. The overall telomere mean lengths and standard deviations were calculated for all 6 cell lines. The CV was then calculated by dividing the standard deviation by the overall telomere mean length. The cell lines are grouped by telomere maintenance mechanism pathways. First is senescent IMR90 cells in purple, followed by two TEL+ cells—UMUC3 in yellow and LNCaP in blue—and lastly, three ALT+ cells: U2OS in green, SK-MEL-2 in red, and Saos-2 in gray. The values adjacent to the ‘X’ located in the center of the box are the mean CVs.

As mentioned above, the higher telomere length heterogeneity in ALT+ cells is evident. Next, we calculated the overall telomere mean length of all telomeres across all chromosome arms (including end telomeres, TFEs, ITS+, and ITS−) and associated standard deviations (Table 1). For example, U2OS and UMUC3 have an overall mean length and standard deviations (STD) of 3.4 kb ± 5.1 kb and 3.1 kb ± 2.8 kb, respectively. ALT+ cells typically have higher STDs. To further quantify the heterogeneity, we calculated the coefficient of variation (CV) of the overall telomere mean length. CV is calculated by dividing the standard deviation by the mean. As shown in Figure 4, the CV values are all above 1 for the three ALT+ cells, while the CVs are all less than 1 for the two TEL+ cells and senescent IMR90, which reflects a much wider distribution of telomere lengths in the three ALT+ cells. Several factors contribute to the elevated CVs in ALT+ cells. First, ALT+ cells have more super-long telomeres. Second, ALT+ cells have increased ITS− and TFEs, whose telomeres are recorded as 0 kb. Finally, telomere measurements in ITS+ are generally shorter compared to end telomeres.

We also investigated the CV values for individual chromosome arms. We calculated the overall telomere mean length of each chromosome arm and its associated standard deviation (Supplementary Table S1, Column F). For example, the overall telomere mean length and standard deviation of 1p for U2OS and UMUC3 are 2.4 ± 2.6 kb and 3.7 ± 3.1 kb, respectively. In U2OS cells, the maximum overall telomere mean length standard deviation is 11.5 kb from 20p while the minimum is 0.0 kb from 3q since all the molecules are fusion/ITS−. The CVs of each chromosome arm for all six cell lines are summarized in Supplementary Table S1. Senescent IMR90 and the two TEL+ cells (UMUC3 and LNCaP) show a narrow distribution with a median CV of less than 1, with two exceptions (8q of UMUC3, CV = 1.36; 14q of IMR90-S, CV = 1.05). In stark contrast, the large majority of chromosome arms of the three ALT+ cells have a much wider CV distribution with median CVs greater than 1.

To compare the heterogeneity of telomeres of the two samples, we performed a two-tailed t-test, concluding that the difference in CV between all combinations of ALT+ and TEL+ cells is statistically significant (Supplementary Table S2). Taken together, our data indicate that telomere length heterogeneity, at both the genome level as well as the individual chromosome arm level, is a very pronounced feature of the ALT+ cells.

## 4. Discussion

Here, we did an in-depth comparative analysis of our previously reported SMTA-OM results from three ALT+ cells (U2OS, Saos-2, and SK-MEL-2), two TEL+ cells (UMUC3 and LNCaP), and the non-transformed but senescent IMR90 (IMR90-S) cells. The results are summarized in Table 1. “N/A” in Table 1 indicates that there are no data relevant to the fusion/ITS+ mean telomere length since there is no fusion/ITS+ molecule detected in the two TEL+ cells and IMR90-S cells. ECTRs were detected and analyzed in the three ALT+ cells but were not detected in the two TEL+ cells and the IMR90-S cells. In addition, there were very few molecules from the short chromosome arms (i.e., the p arms) of all five acrocentric chromosomes (chromosomes 13, 14, 15, 21, 22) that were detected by the SMTA-OM. Therefore, chromosome arms 13p, 14p, 15p, 21p, and 22p were excluded from our analysis for all six cell lines.

Our data indicate that there is no significant difference among the six cell lines for the overall telomere mean lengths except between SK-MEL-2 and IMR90-S (*p =* 0.002) (Supplementary Table S2). The higher prevalence of TFEs and fusion/ITS− found in the ALT+ cells may have obscured the difference. TFEs and fusion/ITS− are recorded as a length of 0 kb in our analysis, which will decrease the overall telomere mean length. In addition, the fusion/ITS+, while it has a measurable telomere signal, tends to be shorter compared to the end telomeres. Therefore, we excluded TFEs and fusions/ITS− when we calculated the end telomere (-TFE) mean length. The end telomere (-TFE) mean length of U2OS is significantly longer than that of UMUC3 and LNCaP (*p* = 0.003, 0.040, respectively) (Supplementary Table S2). The end telomere (-TFE) mean length in SK-MEL-2 is significantly longer compared to IMR90-S cells (*p* = 0.018) and significantly longer in Saos-2 compared to UMUC3 (*p* = 0.028) (Supplementary Table S2). The lack of consistently longer telomeres in ALT+ cells compared to all TEL+ and IMR90-S cells thus leads us to conclude that neither the overall telomere mean length nor the end telomere (-TFE) mean length are reliable readouts for differentiating ALT+ from TEL+ cells.

Here, we reported shorter overall mean telomere lengths in ALT+ cell lines, specifically U2OS, compared to previously published studies [26,27]. For example, U2OS overall telomere mean lengths reported using telomere combing assay (TCA) are between 35–45 kb while measurements with SMTA-OM are 3.4 ± 5.1 kb (Table 1) [27]. Possible reasons for these discrepancies are as follows: First, the SMTA-OM can detect telomeres as short as 100 bp. In our studies we measured 2000 single molecule telomeres from the three ALT+ cell lines, 33% of which had telomeres measuring less than 800 bp. Out of the three ALT+ cell lines analyzed, U2OS had the most telomeres less than 800 bp at 44%, followed by SK-MEL-2 with 30%, and Saos-2 with 25%. The telomeres shorter than 800 bp are most likely missed by the TCA and terminal restriction fragment (TRF) assays [26,27]. Second, the ability of SMTA-OM to detect signal loss from TFEs and ITS− will substantially decrease the overall mean telomere lengths in these ALT+ cell lines. Third, the patterns of the Nt. BspQI nick labeling can unambiguously identify the end of a chromosome arm. This makes it possible to distinguish the fusion/ITS+ from the interstitial telomere repeats found internally, which were excluded from the calculations in the TCA assay [28]. Intriguingly, the telomeres in the fusion/ITS+ are much shorter than the terminal telomeres (Table 1). Fourth, we detected a higher percentage of fusion/ITS+ in the three ALT+ cells: U2OS (23.1%), Saos-2 (17.6%), and SK-MEL-2 (8.6%). These fusions/ITS+ likely render the telomeres in the TRF assay seemingly longer than they really are. Collectively, we believe that SMTA-OM is the best technology to define the features of chromosome ends and telomeres.

The much more prevalent super-long telomeres appear to be a unique feature of the ALT+ cells. The longest telomeres from the two TEL+ cells rarely exceed 10 kb. In contrast, telomeres with a length over 10 kb are easily detected in all three ALT+ cells. In U2OS, SK-MEL-2, and Saos-2, telomeres measuring longer than 10 kb accounted for 69%, 63%, and 43% of chromosome arms, respectively. In stark contrast, UMUC3 and LNCaP had telomeres greater than 10 kb for only 26% and 9% of arms, respectively, while 29% of arms had telomeres longer than 10 kb in IMR90-S cells. The longest telomeres detected in the three ALT+ cells are one molecule of the 16q of Saos-2, measuring at 62.8 kb, one molecule of the 7q of SK-MEL-2 measuring 47.3 kb, and one molecule of the 3p of U2OS measuring at 35.8 kb. We thus propose that the prevalence of super-long telomere can potentially be used to differentiate ALT+ from TEL+ cells.

The telomere lengths of individual chromosome arms vary substantially for both ALT+ and TEL+ cells, which confirmed our previous analysis [20,23]. The SMTA-OM assay can accurately quantify the heterogeneity of telomere length because the telomere length of individual chromosome arms for most chromosomes can be measured at the single-molecule level. In ALT+ cells specifically, there are many super-long telomeres as well as telomeres measuring at 0 kb because of the fusion/ITS− and TFEs. The increased heterogeneity of the telomere length in ALT+ cells is reflected in higher standard deviations and higher CV values (Figure 4). All three ALT+ cells have CV values greater than 1 and a much broader telomere length distribution compared to the two TEL+ cells and IMR90-S cells, which have a much tighter telomere length distribution and CV values less than one.

The percentage of the fusion/ITS+ is also a useful indicator for differentiating ALT+ from TEL+ cells. All three ALT+ cell lines manifest a high prevalence of fusion/ITS+, while no fusion/ITS+ was detected in the two TEL+ cell lines and IMR90-S. The reason for this dramatic difference may be due to heightened spontaneous telomeric damage in the ALT+ cells [29]. When two damaged telomeres are repaired through various DSB repair pathways—for example, nonhomologous end joining (NHEJ) or HDR/BIR—it will lead to end-to-end chromosome fusions (i.e., the dicentric chromosomes), which then manifest as fusion/ITS+ in the SMTA-OM. Intriguingly, there also seem to be chromosome arm-specific effects of the fusion events. Chromosome arms that were analyzed in all three ALT+ cell lines and with detectable fusion/ITS+ include 5q, 7q, 17q, and 21q. There are no shared chromosome arms, of which the fusion/ITS+ is increased in all three ALT+ cells. Finally, there is no fusion/ITS+ detected for 14q in any of the three ALT+ cell lines.

The increase in TFEs is another unique characteristic of the three ALT+ cells, which can also potentially be used as an ALT+ identifier. Similar to fusion/ITS+, significant numbers of TFEs were observed in all three ALT+ cells, while none were detected in the two TEL+ cells and IMR90-S. Similar to the fusion/ITS+, the increased TFEs in the ALT+ cells are likely due to the heightened spontaneous DNA damages in the subtelomeres, which would result in the complete loss of telomeres. U2OS had the greatest elevation of TFEs, followed by SK-MEL-2, and Saos-2 (Table 1). For the individual chromosome arms where measurements could be conducted from U2OS, SK-MEL-2, and Saos-2, TFEs were absent only from 5q. TFEs were observed in all three ALT+ cells from chromosome arms: 1q, 2p, 8q, 9p, 12p, 14q, 16q, 18q, and 19p. Moreover, when two TFEs are repaired by the NHEJ, it would then produce a fusion/ITS− molecule. Alternatively, fusion/ITS− can also be generated through fusing a TFE with a double-stranded DNA break in the internal chromosomal region. Therefore, our data indicate that in addition to the heightened spontaneous DNA damage at telomeres [29], the subtelomeres of the ALT+ cells are also prone to spontaneous DNA damages.

Consistent with the previous findings [15], ECTRs can be easily detected by SMTA-OM and are quite abundant in all three ALT+ cells (Figure 1B). However, the current SMTA-OM assay is unable to differentiate single-stranded DNA molecules from double-stranded DNA molecules. Neither can it distinguish circular DNA from linear DNA. However, the number of ECTRs alone or in combination with other SMTA-OM readouts may be used to distinguish ALT+ cells from the TEL+ cells.

Based on the different SMTA-OM readouts, in addition to the drastic difference between ALT+ and TEL+ cells, there also seem to be distinctive features among the three ALT+ cell lines. For example, U2OS has the highest incidence of fusion/ITS+ and TFEs, followed by Saos-2 and lastly, SK-MEL-2 (Table 1). This order seems to be correlated with the degree of heterogeneity of the overall telomere length as well (Figure 4). U2OS has the broadest CV distribution, with a median CV value of 1.5, followed by Saos-2, with a median CV of 1.4, and lastly SK-MEL-2, with the lowest median CV of 1.2. Far more profound differences can be observed at the individual chromosome arm level among the three ALT+ cells. For example, for the chromosome arm 1p, U2OS and Saos-2 manifested elevated levels of TFEs, fusion/ITS+, and fusion/ITS− percentages, as well as increased CV values, when compared to SK-MEL-2 (Supplementary Table S1, Row 2 Columns R, T, V, F, respectively). A similar trend is also seen with chromosome arm 8q, where Saos-2 and U2OS showed a bigger increase in fusion/ITS+ and TFE percentages, while SK-MEL-2 showed very little to none. Chromosome arm 19q is also very intriguing, being that the super-long telomeres are substantially shorter in U2OS and Saos-2 (5.0 kb and 4.2 kb, respectively) compared to SK-MEL-2 (20.6 kb) while no fusion/ITS− was detected in any of the three ALT+ cells (Supplementary Table S2, Column E). However, fusion/ITS+ and TFEs of 19q were elevated in Saos-2 and U2OS, while very few or none were observed in SK-MEL-2.

## 5. Conclusions

We have previously established a workflow that simultaneously measures and analyzes telomere lengths while optically mapping the subtelomeric/chromosome end regions of long DNA molecules. Three ALT+ cell lines (U2OS, SK-MEL-2, and Saos-2) and two TEL+ cell lines (UMUC3, and LNCaP), along with senescent IMR90, were analyzed for unique indicators for differentiating cancers based on their TMMs. Our analysis of end telomere lengths, heterogeneity of telomere lengths, frequency of fusion/ITS+, fusion/ITS−, super-long telomeres, and TFEs reveals characteristics specific to ALT+ cells. Certain SMTA-OM readouts, alone or in combination with others, can reliably differentiate ALT+ cells from TEL+ cells.

In addition to comparing ALT+ and TEL+ cells, we have also observed significant telomere/chromosome end differences among the three ALT+ cells. The parameters investigated, such as telomere length heterogeneity, ITS+, TFE, and CV, seem to fluctuate even across the three ALT+ cell lines. Saos-2 and U2OS, overall, have similar trends in these parameters compared to SK-MEL-2. When the comparison is conducted at the level of individual chromosome arms, there are even more pronounced discrepancies among the three ALT+ cells. Therefore, these chromosome arm-specific changes detected by the SMTA-OM may also be used to differentiate ALT+ cancers.

Along with two TEL+ cell lines, (UMUC3 and LNCaP) and the senescent IMR90 cells, we only analyzed three ALT+ cell lines (U2OS, SK-MEL-2, and Saos-2). Although certain SMTA-OM readouts do manifest pronounced differences among the three ALT+ cells, analysis of additional ALT+ cell lines is warranted to further confirm SMTA-OM’s capacity to discern between different subtypes of ALT+ cancer cells.

In recent years, a few groups, including ours, identified FANCM as a promising molecular target for treating ALT+ cancers [11,12,30]. Another recent study reported that the ALT+ cells are especially sensitive to the treatment of small molecule inhibitors targeting ATR (ATRi) and PARP (PARPi) [31]. Four PARPi have been approved by the FDA to treat various *BRCA1*/*2*-deficient cancers [32]. A few ATRi are currently in various stages of clinical trials [33,34]. We propose that in the near future, SMTA-OM can potentially be used in the clinic as a diagnostic tool to reliably identify ALT+ tumors for targeted cancer therapy.

## Figures and Tables

**Table 1:** Summary of SMTA-OM readouts for ALT+ (U2OS, Saos-2, SK-MEL-2), TEL+ (UMUC3, LNCaP), and IMR90-S cells. It includes overall telomere mean length, end telomere mean length excluding the telomere-free ends (-TFE), fusion/internal telomere-like signal (ITS+), mean telomere length, heterogeneity of telomere length represented by the coefficient of variation (CV), the percentage of fusion/ITS+ molecules, the percentage of fusion/internal telomere-like signal loss (ITS−) molecules, the percentage of telomere-free ends (TFEs), and the status of the extrachromosomal telomeric repeats (ECTR). N/A indicates no fusion/ITS+ observed in a cell line.

	ALT+			TEL+		Control
	U2OS	Saos-2	SK-MEL-2	UMUC3	LNCaP	IMR90-S
Overall telomere mean length (kb)	3.4 ± 5.1	3.8 ± 5.2	3.2 ± 3.8	3.1 ± 2.8	3.2 ± 2.2	4.0 ± 2.6
End telomere (-TFEs) mean length (kb)	5.0 ± 2.2	4.5 ± 5.6	3.5 ± 3.9	3.1 ± 2.8	3.2 ± 2.1	4.0 ± 2.6
Fusion/ITS+ mean telomere length (kb)	1.8 ± 3.3	2.7 ± 3.6	3.7 ± 3.4	N/A	N/A	N/A
The heterogeneity of telomere length (CV)	1.4	1.2	1.1	0.8	0.5	0.6
Fusion/ITS+ (%)	23.1	17.6	8.6	0	0	0
Fusion/ITS− (%)	12.2	0.5	0.5	0	0	0
Telomere Free Ends (TFEs) (%)	6.4	6.3	7.6	0	0	0
ECTR	High	High	High	No data	No data	No data

## Data Availability

The BioNano whole-genome mapping data from this study have been submitted to the NCBI BioProject (https://www.ncbi.nlm.nih.gov/bioproject/396850 (accessed on 8 August 2022)) under accession number PRJNA396850. The BioNano supplemental data at this accession refer to SK-MEL-2 (SUPPF_0000003660), Saos-2 (SUPPF_0000003661), U2OS (SUPPF_0000003665), IMR90 (SUPPF_0000001237), LNCaP (SUPPF_0000001236), and UMUC3 (SUPPF_0000001235). The hg38 reference used was from the Genome Reference Consortium, the December 2013 version GRCh38/hg38 GCA_000001405.15, and downloaded from the UCSC Genome Browser, at http://hgdownload.soe.ucsc.edu/goldenPath/hg38/chromosomes (accessed on 8 August 2022).

## Supplementary Materials

The following supporting information can be downloaded at: https://www.mdpi.com/article/10.3390/genes14061278/s1, Table S1: Calculations; Table S2: *p* values.
