# Supplementary material for: Single-Molecule Telomere Assay via Optical Mapping (SMTA-OM) Can Potentially Define the ALT Positivity of Cancer
Source: Genes (Basel). 2023 Jun 16;14(6):1278. doi: 10.3390/genes14061278 (PMC10297966; doi:10.3390/genes14061278)
Supplement: Supplementary file 1 [file genes-14-01278-s001.zip › Supplementary Table S1_Calculations.pdf]

A	B	C	D	E	F	G	H	I	J	K	L	M	N	O	P	Q	R	S	T	U	V
**U2OS**																					
**Chr arm**	**Overall counts**	**Overall Mean**	**Overall STD**	**Overall Max**	**CV**	**EndTel % (-TFE)**	**EndTel Counts**	**EndTel Counts (-TFE)**	**EndTel Mean (-TFE)**	**EndTel STDev (-TFE)**	**EndTel Max**	**TFE counts**	**TFE %**	**ITS Counts**	**ITS Mean**	**ITS STDev**	**ITS %**	**ITS+ counts**	**ITS+ %**	**ITS- counts**	**ITS- %**
1p	24	2.40	2.6	7.4	1.1	58.3	15	14	2.9	2.2	6.2	1	4.2	9	1.9	3.2	37.5	4	16.7	5	20.8
1q	29	1.19	2.2	9.4	1.9	20.7	7	6	4.0	3.9	9.4	1	3.4	22	0.5	0.6	75.9	18	62.1	4	13.8
2p	20	2.34	3.0	10.7	1.3	60.0	14	12	2.5	2.5	7.0	2	10.0	6	2.9	4.3	30.0	4	20.0	2	10.0
2q	38	5.08	5.6	23.8	1.1	92.1	35	35	5.5	5.7	23.8	0	0.0	3	0.4	0.6	7.9	1	2.6	2	5.3
3p	23	5.50	7.4	35.8	1.3	69.6	16	16	6.6	8.4	35.8	0	0.0	7	2.9	3.8	30.4	7	30.4	0	0.0
3q	23	0.00	0.0	0.0	0.0	0.0	7	0	0.0	0.0	0.0	7	30.4	16	0.0	0.0	69.6	0	0.0	16	69.6
4p	22	2.92	3.3	11.8	1.1	86.4	19	19	3.2	3.5	11.8	0	0.0	3	1.3	1.9	13.6	2	9.1	1	4.5
5p	36	2.53	3.9	15.5	1.5	58.3	24	21	4.3	4.2	15.5	3	8.3	12	0.0	0.1	33.3	9	25.0	3	8.3
5q	36	5.56	5.3	24.2	0.9	91.7	33	33	5.5	5.4	24.2	0	0.0	3	6.6	3.7	8.3	3	8.3	0	0.0
6p	14	1.87	1.9	6.0	1.0	57.1	8	8	2.8	1.8	6.0	0	0.0	6	0.6	1.1	42.9	6	42.9	0	0.0
6q	23	7.50	7.6	28.2	1.0	91.3	21	21	7.8	7.9	28.2	0	0.0	2	4.8	1.5	8.7	2	8.7	0	0.0
7p	22	1.14	3.3	15.8	2.9	22.7	5	5	3.4	6.9	15.8	0	0.0	17	0.5	0.4	77.3	17	77.3	0	0.0
7q	13	5.36	4.4	11.8	0.8	84.6	11	11	4.9	4.4	11.7	0	0.0	2	8.0	5.4	15.4	2	15.4	0	0.0
8p	11	2.07	3.0	8.4	1.5	72.7	8	8	2.7	3.3	8.4	0	0.0	3	0.3	0.2	27.3	3	27.3	0	0.0
8q	49	0.99	2.3	12.9	2.3	34.7	23	17	2.4	3.5	12.9	6	12.2	26	0.3	0.3	53.1	19	38.8	7	14.3
9p	17	2.77	5.1	17.8	1.8	41.2	9	7	3.3	4.9	12.2	2	11.8	8	3.0	6.1	47.1	5	29.4	3	17.6
9q	31	4.46	4.9	17.7	1.1	74.2	23	23	5.2	5.3	17.7	0	0.0	8	2.5	2.6	25.8	6	19.4	2	6.5
10q	4	1.93	3.8	7.7	2.0	50.0	2	2	3.9	5.4	7.7	0	0.0	2	0.0	0.0	50.0	0	0.0	2	50.0
11p	25	2.19	4.1	18.1	1.9	48.0	12	12	4.4	5.1	18.1	0	0.0	13	0.2	0.3	52.0	3	12.0	10	40.0
11q	13	2.63	3.5	9.5	1.4	53.8	8	7	4.8	3.7	9.5	1	7.7	5	0.1	0.2	38.5	4	30.8	1	7.7
12p	34	3.27	5.4	27.6	1.6	58.8	22	20	4.2	3.3	11.1	2	5.9	12	2.3	8.0	35.3	1	2.9	11	32.4
12q	32	2.93	4.2	16.6	1.4	56.3	22	18	4.4	4.7	16.6	4	12.5	10	0.5	0.9	31.3	7	21.9	3	9.4
13q	32	5.65	5.4	20.9	1.0	93.8	30	30	5.5	5.4	20.9	0	0.0	2	8.6	5.8	6.3	2	6.3	0	0.0
14q	20	1.70	4.4	18.7	2.6	30.0	16	6	5.7	6.8	18.7	10	50.0	4	0.0	0.0	20.0	0	0.0	4	20.0
15q	30	3.70	5.3	18.5	1.4	56.7	23	17	6.5	5.7	18.5	6	20.0	7	0.2	0.4	23.3	3	10.0	4	13.3
16q	39	5.17	5.1	17.1	1.0	71.8	32	28	6.1	5.3	17.1	4	10.3	7	4.5	4.2	17.9	7	17.9	0	0.0
17q	35	5.01	5.2	18.2	1.0	85.7	30	30	5.4	5.3	18.2	0	0.0	5	2.4	4.4	14.3	2	5.7	3	8.6
18p	13	3.16	4.8	16.6	1.5	69.2	10	9	4.6	5.2	16.6	1	7.7	3	0.0	0.0	23.1	0	0.0	3	23.1
18q	40	0.71	0.8	2.9	1.1	12.5	9	5	0.9	1.2	2.9	4	10.0	31	0.9	0.7	77.5	26	65.0	5	12.5
19p	32	6.80	7.9	35.9	1.2	93.8	31	30	7.2	8.1	35.9	1	3.1	1	3.2	0.0	3.1	1	3.1	0	0.0
19q	26	1.43	1.1	5.0	0.8	0.0	1	0	0.0	0.0	0.0	1	3.8	25	1.5	1.1	96.2	25	96.2	0	0.0
20p	23	7.04	11.5	47.5	1.6	87.0	20	20	7.8	12.2	47.5	0	0.0	3	2.3	2.1	13.0	2	8.7	1	4.3
20q	24	3.16	3.8	14.2	1.2	87.5	21	21	3.4	3.9	14.2	0	0.0	3	1.3	1.1	12.5	3	12.5	0	0.0
21q	43	2.04	3.9	16.2	1.9	27.9	13	12	5.3	4.7	16.2	1	2.3	30	0.9	2.8	69.8	13	30.2	17	39.5

SK-MEL-2

A	B	C	D	E	F	G	H	I	J	K	L	M	N	O	P	Q	R	S	T	U	V
Chr arm	Overall counts	Overall Mean	Overall STD	Overall Max	CV	EndTel % (-TFE)	EndTel Counts	EndTel (-TFE) counts	EndTel Mean (-TFE)	EndTel STDev (-TFE)	EndTel Max	TFE counts	TFE %	ITS Counts	ITS Mean	ITS STDev	ITS %	ITS+ counts	ITS+ %	ITS- counts	ITS- %
1p	22	4.5	3.4	12.2	0.8	100.0	22	22	4.49	3.4	12.2	0	0.0	0	0.0	0.0	0.0	0	0.0	0	0.0
1q	30	4.2	3.4	12.0	0.8	80.0	25	24	4.47	3.6	12.0	1	3.3	5	3.9	2.0	16.7	5	16.7	0	0.0
2p	34	3.1	4.0	18.9	1.3	94.1	34	32	3.26	4.1	18.9	2	5.9	0	0.0	0.0	0.0	0	0.0	0	0.0
2q	35	2.7	2.4	8.6	0.9	88.6	32	31	2.67	2.4	8.6	1	2.9	3	3.6	1.1	8.6	3	8.6	0	0.0
3p	25	4.2	3.6	14.7	0.9	100.0	25	25	4.23	3.6	14.7	0	0.0	0	0.0	0.0	0.0	0	0.0	0	0.0
3q	8	2.6	6.5	18.7	2.5	0.0	0	0	0.00	0.0	0.0	0	0.0	8	2.6	6.5	100.0	4	50.0	4	50.0
4p	36	2.1	3.0	9.8	1.4	100.0	36	36	2.05	3.0	9.8	0	0.0	0	0.0	0.0	0.0	0	0.0	0	0.0
5q	38	4.0	3.3	14.4	0.8	97.4	37	37	4.07	3.3	14.4	0	0.0	1	2.4	0.0	2.6	1	2.6	0	0.0
6q	25	3.1	3.6	13.9	1.2	76.0	25	19	4.05	3.7	13.9	6	24.0	0	0.0	0.0	0.0	0	0.0	0	0.0
7p	14	1.7	1.9	5.7	1.2	85.7	14	12	1.95	2.0	5.7	2	14.3	0	0.0	0.0	0.0	0	0.0	0	0.0
7q	43	4.1	7.4	47.3	1.8	81.4	37	35	4.26	8.1	47.3	2	4.7	6	4.7	2.3	14.0	6	14.0	0	0.0
8p	42	2.8	3.0	11.7	1.1	85.7	41	36	2.95	2.9	11.7	5	11.9	1	10.3	0.0	2.4	1	2.4	0	0.0
8q	37	4.1	4.0	14.2	1.0	97.3	37	36	4.21	4.0	14.2	1	2.7	0	0.0	0.0	0.0	0	0.0	0	0.0
9p	37	2.9	3.1	14.1	1.1	83.8	32	31	3.06	3.2	14.1	1	2.7	5	2.6	2.5	13.5	5	13.5	0	0.0
9q	20	1.1	1.6	4.4	1.5	80.0	20	16	1.32	1.7	4.4	4	20.0	0	0.0	0.0	0.0	0	0.0	0	0.0
10p	26	3.2	3.2	11.2	1.0	88.5	25	23	3.46	3.2	11.2	2	7.7	1	2.9	0.0	3.8	1	3.8	0	0.0
10q	22	3.9	4.4	13.7	1.1	86.4	20	19	3.74	4.2	13.7	1	4.5	2	7.8	6.8	9.1	2	9.1	0	0.0
11q	10	3.0	4.7	13.2	1.6	60.0	10	6	4.99	5.2	13.2	4	40.0	0	0.0	0.0	0.0	0	0.0	0	0.0
12p	22	2.7	2.0	7.2	0.8	81.8	20	18	2.84	1.6	6.4	2	9.1	2	4.0	4.5	9.1	2	9.1	0	0.0
12q	31	4.8	4.3	13.7	0.9	93.5	31	29	5.17	4.3	13.7	2	6.5	0	0.0	0.0	0.0	0	0.0	0	0.0
13q	30	2.4	3.0	9.3	1.3	90.0	30	27	2.64	3.1	9.3	3	10.0	0	0.0	0.0	0.0	0	0.0	0	0.0
14q	25	1.5	2.9	14.3	2.0	84.0	25	21	1.73	3.1	14.3	4	16.0	0	0.0	0.0	0.0	0	0.0	0	0.0
15q	30	4.8	3.5	14.3	0.7	96.7	30	29	4.94	3.5	14.3	1	3.3	0	0.0	0.0	0.0	0	0.0	0	0.0
16q	24	1.7	2.2	10.9	1.3	75.0	20	18	1.35	1.1	3.5	2	8.3	4	4.4	4.6	16.7	4	16.7	0	0.0
17q	29	3.1	4.2	21.0	1.4	89.7	28	26	3.22	4.4	21.0	2	6.9	1	4.9	0.0	3.4	1	3.4	0	0.0
18p	4	0.6	0.4	0.8	0.8	50.0	2	2	0.6	0.4	1.1	0	0.0	2	0.8	0.2	50.0	1	25.0	1	25.0
18q	36	4.1	3.3	12.2	0.8	80.6	30	29	4.04	3.3	12.2	1	2.8	6	4.9	2.9	16.7	6	16.7	0	0.0
19p	36	2.9	3.2	13.2	1.1	83.3	31	30	2.66	3.0	13.2	1	2.8	5	5.1	3.9	13.9	5	13.9	0	0.0
19q	32	5.0	5.3	20.6	1.1	93.8	30	30	5.11	5.4	20.6	0	0.0	2	2.6	2.3	6.3	2	6.3	0	0.0
20p	20	4.5	4.7	16.4	1.0	75.0	16	15	4.84	5.2	16.4	1	5.0	4	4.6	2.5	20.0	4	20.0	0	0.0
20q	45	1.5	2.6	16.5	1.8	53.3	27	24	1.82	3.3	16.5	3	6.7	18	1.2	1.3	40.0	17	37.8	1	2.2
21q	34	4.3	3.7	15.3	0.9	88.2	30	30	4.14	3.8	15.3	0	0.0	4	5.1	2.4	11.8	4	11.8	0	0.0
XqYq	21	1.4	1.8	7.5	1.3	66.7	17	14	1.38	1.9	7.5	3	14.3	4	2.6	1.3	19.0	4	19.0	0	0.0

A	B	C	D	E	F	G	H	I	J	K	L	M	N	O	P	Q	R	S	T	U	V

Saos-2

Chr arm	Overall counts	Overall Mean	Overall STD	Overall Max	CV	EndTel % (-TFE)	EndTel Counts	EndTel Counts (-TFE)	EndTel Mean (-TFE)	EndTel STDev (-TFE)	EndTel Max	TFE counts	TFE %	ITS Counts	ITS Mean	ITS STDev	ITS %	ITS + counts	ITS+ %	ITS- counts	ITS- %
1p	35	2.2	5.3	30.1	2.3	28.6	13	10	5.8	9.1	30.1	3	8.6	22	0.9	0.9	62.9	19.0	54.3	3	8.571
1q	7	1.7	2.1	5.3	1.3	0.0	3	0	0.0	0.0	0.0	3	42.9	4	2.9	2.0	57.1	4.0	57.1	0	0
2p	30	4.9	4.1	12.9	0.8	96.7	30	29	5.1	4.1	12.9	1	3.3	0	0.0	0.0	0.0	0.0	0.0	0	0
3p	31	7.5	5.6	22.9	0.8	96.8	31	30	7.8	5.6	22.9	1	3.2	0	0.0	0.0	0.0	0.0	0.0	0	0
3q	8	0.8	0.7	1.9	0.8	0.0	1	0	0.0	0.0	0.0	1	12.5	7	0.9	0.7	87.5	7.0	87.5	0	0
4p	23	3.4	3.4	12.5	1.0	91.3	23	21	3.7	3.3	12.5	2	8.7	0	0.0	0.0	0.0	0.0	0.0	0	0
4q	4	0.6	0.8	1.8	1.4	75.0	4	3	0.8	0.9	1.8	1	25.0	0	0.0	0.0	0.0	0.0	0.0	0	0
5p	21	4.6	4.8	19.2	1.0	61.9	13	13	5.8	5.6	19.2	0	0.0	8	2.7	1.9	38.1	8.0	38.1	0	0
5q	27	6.3	4.4	15.5	0.7	96.3	26	26	6.5	4.4	15.5	0	0.0	1	1.6	0.0	3.7	1.0	3.7	0	0
6p	15	7.3	9.5	27.5	1.3	86.7	14	13	8.2	10.0	27.5	1	6.7	1	3.1	0.0	6.7	1.0	6.7	0	0
6q	7	10.4	7.7	19.8	0.7	0.0	2	0	0.0	0.0	0.0	2	28.6	5	14.5	3.7	71.4	5.0	71.4	0	0
7q	27	2.0	1.5	5.5	0.7	96.3	26	26	2.1	1.5	5.5	0	0.0	1	1.3	0.0	3.7	1.0	3.7	0	0
8q	32	1.4	1.0	4.3	0.7	40.6	14	13	1.3	0.8	3.0	1	3.1	18	1.6	1.1	56.3	18.0	56.3	0	0
9p	25	4.9	5.3	17.8	1.1	96.0	25	24	5.1	5.4	17.8	1	4.0	0	0.0	0.0	0.0	0.0	0.0	0	0
10p	19	2.1	1.9	6.8	0.9	89.5	19	17	2.3	1.9	6.8	2	10.5	0	0.0	0.0	0.0	0.0	0.0	0	0
10q	32	2.9	3.8	16.1	1.3	93.8	30	30	3.0	3.9	16.1	0	0.0	2	0.7	0.8	6.3	2.0	6.3	0	0
11p	11	5.5	5.2	13.6	1.0	54.5	6	6	6.2	5.6	13.6	0	0.0	5	4.7	5.2	45.5	5.0	45.5	0	0
12p	27	6.3	4.8	15.0	0.8	92.6	27	25	6.8	4.6	15.0	2	7.4	0	0.0	0.0	0.0	0.0	0.0	0	0
13q	35	6.5	6.1	23.3	0.9	82.9	30	29	6.6	6.2	23.3	1	2.9	5	7.6	6.2	14.3	5.0	14.3	0	0
14q	24	2.2	2.9	7.9	1.3	95.8	24	23	2.3	2.9	7.9	1	4.2	0	0.0	0.0	0.0	0.0	0.0	0	0
16q	12	6.8	17.7	62.8	2.6	83.3	12	10	8.2	19.2	62.8	2	16.7	0	0.0	0.0	0.0	0.0	0.0	0	0
17q	23	1.7	2.3	10.2	1.3	21.7	7	5	2.6	1.7	4.8	2	8.7	16	1.7	2.5	69.6	16.0	69.6	0	0
18p	30	3.8	3.7	16.2	1.0	100.0	30	30	3.8	3.7	16.2	0	0.0	0	0.0	0.0	0.0	0.0	0.0	0	0
18q	28	2.4	3.4	14.2	1.4	85.7	28	24	2.8	3.5	14.2	4	14.3	0	0.0	0.0	0.0	0.0	0.0	0	0
19p	6	4.3	4.0	11.7	0.9	0.0	1	0	0.0	0.0	0.0	1	16.7	5	5.1	3.9	83.3	5.0	83.3	0	0
19q	14	0.4	1.1	4.2	3.1	0.0	12	0	0.0	0.0	0.0	12	85.7	2	2.6	2.3	14.3	2.0	14.3	0	0
20p	21	1.9	1.5	4.9	0.8	95.2	21	20	2.0	1.5	4.9	1	4.8	0	0.0	0.0	0.0	0.0	0.0	0	0
21q	29	1.7	0.8	3.6	0.5	20.7	7	6	1.3	0.6	2.1	1	3.4	22	1.9	0.7	75.9	22.0	75.9	0	0

UMUC3

A	B	C	D	E	F	G	H	I	J	K	L	M	N	O	P	Q	R	S	T	U	V
**Chr arm**	**Overall counts**	**Overall Mean**	**Overall STD**	**Overall Max**	**CV**	**EndTel % (-TFE)**	**EndTel counts**	**EndTel (-TFE) counts**	**EndTel Mean**	**EndTel STDev**	**EndTel Max**	**TFE counts**	**TFE %**	**ITS counts**	**ITS Mean**	**ITS STDev**	**ITS %**	**ITS+ counts**	**ITS+ %**	**ITS- counts**	**ITS- %**
1p	30	3.7	3.1	10.8	0.8	100.0	30	30.0	3.7	3.1	10.8	0	0.0	0.0	0.0	0.0	0.0	0	0.0	0	0.0
1q	25	3.0	2.9	11.3	1.0	100.0	25	25.0	3.0	2.9	11.3	0	0.0	0.0	0.0	0.0	0.0	0	0.0	0	0.0
2p	30	2.4	2.0	6.7	0.8	100.0	30	30.0	2.4	2.0	6.7	0	0.0	0.0	0.0	0.0	0.0	0	0.0	0	0.0
2q	30	2.4	2.0	7.8	0.8	100.0	30	30.0	2.4	2.0	7.8	0	0.0	0.0	0.0	0.0	0.0	0	0.0	0	0.0
3p	30	5.4	3.5	14.6	0.7	100.0	30	30.0	5.4	3.5	14.6	0	0.0	0.0	0.0	0.0	0.0	0	0.0	0	0.0
3q	30	4.6	3.3	12.0	0.7	100.0	30	30.0	4.6	3.3	12.0	0	0.0	0.0	0.0	0.0	0.0	0	0.0	0	0.0
4p	30	3.7	2.9	9.7	0.8	100.0	30	30.0	3.7	2.9	9.7	0	0.0	0.0	0.0	0.0	0.0	0	0.0	0	0.0
4q	9	4.0	3.3	9.6	0.8	100.0	9	9.0	4.0	3.3	9.6	0	0.0	0.0	0.0	0.0	0.0	0	0.0	0	0.0
5p	20	1.8	1.4	5.6	0.8	100.0	20	20.0	1.8	1.4	5.6	0	0.0	0.0	0.0	0.0	0.0	0	0.0	0	0.0
5q	30	2.9	1.9	7.3	0.7	100.0	30	30.0	2.9	1.9	7.3	0	0.0	0.0	0.0	0.0	0.0	0	0.0	0	0.0
6q	23	4.5	3.3	9.7	0.7	100.0	23	23.0	4.5	3.3	9.7	0	0.0	0.0	0.0	0.0	0.0	0	0.0	0	0.0
7q	30	2.7	2.0	8.1	0.7	100.0	30	30.0	2.7	2.0	8.1	0	0.0	0.0	0.0	0.0	0.0	0	0.0	0	0.0
8q	30	1.0	1.3	6.7	1.4	100.0	30	30.0	1.0	1.3	6.7	0	0.0	0.0	0.0	0.0	0.0	0	0.0	0	0.0
9p	30	1.7	1.7	5.8	1.0	100.0	30	30.0	1.7	1.7	5.8	0	0.0	0.0	0.0	0.0	0.0	0	0.0	0	0.0
9q	30	1.9	1.0	4.5	0.5	100.0	30	30.0	1.9	1.0	4.5	0	0.0	0.0	0.0	0.0	0.0	0	0.0	0	0.0
11q	20	3.5	3.5	12.7	1.0	100.0	20	20.0	3.5	3.5	12.7	0	0.0	0.0	0.0	0.0	0.0	0	0.0	0	0.0
12p	30	3.7	3.2	14.1	0.9	100.0	30	30.0	3.7	3.2	14.1	0	0.0	0.0	0.0	0.0	0.0	0	0.0	0	0.0
12q	30	5.8	3.8	14.2	0.6	100.0	30	30.0	5.8	3.8	14.2	0	0.0	0.0	0.0	0.0	0.0	0	0.0	0	0.0
13q	30	2.5	2.4	10.0	0.9	100.0	30	30.0	2.5	2.4	10.0	0	0.0	0.0	0.0	0.0	0.0	0	0.0	0	0.0
14q	9	1.9	1.5	4.3	0.8	100.0	9	9.0	1.9	1.5	4.3	0	0.0	0.0	0.0	0.0	0.0	0	0.0	0	0.0
15q	18	2.9	2.2	8.1	0.7	100.0	18	18.0	2.9	2.2	8.1	0	0.0	0.0	0.0	0.0	0.0	0	0.0	0	0.0
18p	19	3.5	3.1	11.3	0.9	100.0	19	19.0	3.5	3.1	11.3	0	0.0	0.0	0.0	0.0	0.0	0	0.0	0	0.0
18q	30	3.0	1.9	8.8	0.6	100.0	30	30.0	3.0	1.9	8.8	0	0.0	0.0	0.0	0.0	0.0	0	0.0	0	0.0
19p	30	2.1	0.8	4.0	0.4	100.0	30	30.0	2.1	0.8	4.0	0	0.0	0.0	0.0	0.0	0.0	0	0.0	0	0.0
20p	30	1.6	1.1	5.0	0.7	100.0	30	30.0	1.6	1.1	5.0	0	0.0	0.0	0.0	0.0	0.0	0	0.0	0	0.0
20q	30	2.1	2.1	7.0	1.0	100.0	30	30.0	2.1	2.1	7.0	0	0.0	0.0	0.0	0.0	0.0	0	0.0	0	0.0
21q	30	3.2	2.6	10.5	0.8	100.0	30	30.0	3.2	2.6	10.5	0	0.0	0.0	0.0	0.0	0.0	0	0.0	0	0.0
XqYq	16	4.9	4.7	14.7	1.0	100.0	16	16.0	4.9	4.7	14.7	0	0.0	0.0	0.0	0.0	0.0	0	0.0	0	0.0

A	B	C	D	E	F	G	H	I	J	K	L	M	N	O	P	Q	R	S	T	U	V
**LNCaP**																					
**Chr arm**	**Overall counts**	**Overall Mean**	**Overall STD**	**Overall Max**	**CV**	**EndTel % (-TFE)**	**EndTel counts**	**EndTel (-TFE) counts**	**EndTel Mean**	**EndTel STDev**	**EndTel Max**	**TFE counts**	**TFE %**	**ITS counts**	**ITS Mean**	**ITS STDev**	**ITS %**	**ITS+ counts**	**ITS+ %**	**ITS- counts**	**ITS- %**
1p	12	13.2	5.4	23.5	0.4	100.0	12	12	13.2	5.4	23.5	0	0.0	0.0	0.0	0.0	0.0	0	0.0	0	0.0
1q	30	3.2	1.2	5.7	0.4	100.0	30	30	3.2	1.2	5.7	0	0.0	0.0	0.0	0.0	0.0	0	0.0	0	0.0
2p	30	3.1	1.2	7.5	0.4	100.0	30	30	3.1	1.2	7.5	0	0.0	0.0	0.0	0.0	0.0	0	0.0	0	0.0
2q	30	3.3	2.0	7.8	0.6	100.0	30	30	3.3	2.0	7.8	0	0.0	0.0	0.0	0.0	0.0	0	0.0	0	0.0
3p	30	3.5	1.7	7.7	0.5	100.0	30	30	3.5	1.7	7.7	0	0.0	0.0	0.0	0.0	0.0	0	0.0	0	0.0
3q	29	3.6	2.1	8.9	0.6	100.0	29	29	3.6	2.1	8.9	0	0.0	0.0	0.0	0.0	0.0	0	0.0	0	0.0
4p	30	3.3	1.7	8.4	0.5	100.0	30	30	3.3	1.7	8.4	0	0.0	0.0	0.0	0.0	0.0	0	0.0	0	0.0
5p	28	3.4	1.5	7.2	0.4	100.0	28	28	3.4	1.5	7.2	0	0.0	0.0	0.0	0.0	0.0	0	0.0	0	0.0
5q	30	4.5	1.6	9.2	0.4	100.0	30	30	4.5	1.6	9.2	0	0.0	0.0	0.0	0.0	0.0	0	0.0	0	0.0
6p	30	3.1	1.8	7.9	0.6	100.0	30	30	3.1	1.8	7.9	0	0.0	0.0	0.0	0.0	0.0	0	0.0	0	0.0
6q	23	4.1	1.6	7.4	0.4	100.0	23	23	4.1	1.6	7.4	0	0.0	0.0	0.0	0.0	0.0	0	0.0	0	0.0
7p	30	2.5	1.0	5.1	0.4	100.0	30	30	2.5	1.0	5.1	0	0.0	0.0	0.0	0.0	0.0	0	0.0	0	0.0
7q	30	2.7	1.4	6.3	0.5	100.0	30	30	2.7	1.4	6.3	0	0.0	0.0	0.0	0.0	0.0	0	0.0	0	0.0
8p	30	2.3	1.3	5.1	0.6	100.0	30	30	2.3	1.3	5.1	0	0.0	0.0	0.0	0.0	0.0	0	0.0	0	0.0
8q	30	2.3	1.6	7.1	0.7	100.0	30	30	2.3	1.6	7.1	0	0.0	0.0	0.0	0.0	0.0	0	0.0	0	0.0
9p	30	3.4	2.9	14.5	0.8	100.0	30	30	3.4	2.9	14.5	0	0.0	0.0	0.0	0.0	0.0	0	0.0	0	0.0
9q	30	3.3	1.5	6.7	0.5	100.0	30	30	3.3	1.5	6.7	0	0.0	0.0	0.0	0.0	0.0	0	0.0	0	0.0
10p	25	2.3	1.6	7.1	0.7	100.0	25	25	2.3	1.6	7.1	0	0.0	0.0	0.0	0.0	0.0	0	0.0	0	0.0
10q	16	2.0	0.8	3.4	0.4	100.0	16	16	2.0	0.8	3.4	0	0.0	0.0	0.0	0.0	0.0	0	0.0	0	0.0
11p	25	2.6	1.0	5.1	0.4	100.0	25	25	2.6	1.0	5.1	0	0.0	0.0	0.0	0.0	0.0	0	0.0	0	0.0
11q	30	2.2	1.5	7.6	0.7	100.0	30	30	2.2	1.5	7.6	0	0.0	0.0	0.0	0.0	0.0	0	0.0	0	0.0
12p	30	3.0	2.3	13.2	0.7	100.0	30	30	3.0	2.3	13.2	0	0.0	0.0	0.0	0.0	0.0	0	0.0	0	0.0
12q	30	3.2	1.9	8.1	0.6	100.0	30	30	3.2	1.9	8.1	0	0.0	0.0	0.0	0.0	0.0	0	0.0	0	0.0
13q	25	3.0	1.9	6.8	0.6	100.0	25	25	3.0	1.9	6.8	0	0.0	0.0	0.0	0.0	0.0	0	0.0	0	0.0
14q	13	3.9	2.0	7.5	0.5	100.0	13	13	3.9	2.0	7.5	0	0.0	0.0	0.0	0.0	0.0	0	0.0	0	0.0
15q	30	2.8	1.1	5.4	0.4	100.0	30	30	2.8	1.1	5.4	0	0.0	0.0	0.0	0.0	0.0	0	0.0	0	0.0
16p	31	4.0	2.4	10.3	0.6	100.0	31	31	4.0	2.4	10.3	0	0.0	0.0	0.0	0.0	0.0	0	0.0	0	0.0
16q	30	3.0	0.9	5.1	0.3	100.0	30	30	3.0	0.9	5.1	0	0.0	0.0	0.0	0.0	0.0	0	0.0	0	0.0
17q	30	2.6	1.0	5.1	0.4	100.0	30	30	2.6	1.0	5.1	0	0.0	0.0	0.0	0.0	0.0	0	0.0	0	0.0
18q	28	4.1	1.7	9.3	0.4	100.0	28	28	4.1	1.7	9.3	0	0.0	0.0	0.0	0.0	0.0	0	0.0	0	0.0
19p	30	2.7	1.5	7.7	0.6	100.0	30	30	2.7	1.5	7.7	0	0.0	0.0	0.0	0.0	0.0	0	0.0	0	0.0
20p	15	1.9	1.0	3.5	0.5	100.0	15	15	1.9	1.0	3.5	0	0.0	0.0	0.0	0.0	0.0	0	0.0	0	0.0
20q	30	2.7	1.4	6.2	0.5	100.0	30	30	2.7	1.4	6.2	0	0.0	0.0	0.0	0.0	0.0	0	0.0	0	0.0
21q	29	3.9	1.7	7.3	0.4	100.0	29	29	3.9	1.7	7.3	0	0.0	0.0	0.0	0.0	0.0	0	0.0	0	0.0

A B C D E F G H I J K L M N O P Q R S T U V

IMR90-S

Chr arm	Overall counts	Overall Mean	Overall STD	Overall Max	CV	EndTel % (-TFE)	EndTel counts	EndTel (-TFE) counts	EndTel Mean	EndTel STDev	EndTel Max	TFE counts	TFE %	ITS counts	ITS Mean	ITS STDev	ITS %	ITS+ counts	ITS+ %	ITS- counts	ITS- %
1q	22	4.8	2.1	9.1	0.4	100.0	22	22	4.8	2.1	9.1	0	0.00	0	0.0	0.0	0.0	0	0.0	0	0.0
2p	28	3.2	2.1	9.1	0.7	100.0	28	28	3.2	2.1	9.1	0	0.00	0	0.0	0.0	0.0	0	0.0	0	0.0
2q	30	5.5	4.8	16.4	0.9	100.0	30	30	5.5	4.8	16.4	0	0.00	0	0.0	0.0	0.0	0	0.0	0	0.0
3p	30	4.9	2.7	15.7	0.6	100.0	30	30	4.9	2.7	15.7	0	0.00	0	0.0	0.0	0.0	0	0.0	0	0.0
4p	28	4.8	2.8	10.9	0.6	100.0	28	28	4.8	2.8	10.9	0	0.00	0	0.0	0.0	0.0	0	0.0	0	0.0
5p	26	3.4	2.3	7.4	0.7	100.0	26	26	3.4	2.3	7.4	0	0.00	0	0.0	0.0	0.0	0	0.0	0	0.0
5q	30	3.8	2.2	8.6	0.6	100.0	30	30	3.8	2.2	8.6	0	0.00	0	0.0	0.0	0.0	0	0.0	0	0.0
6p	28	3.2	1.5	5.6	0.5	100.0	28	28	3.2	1.5	5.6	0	0.00	0	0.0	0.0	0.0	0	0.0	0	0.0
6q	26	4.2	2.8	10.5	0.7	100.0	26	26	4.2	2.8	10.5	0	0.00	0	0.0	0.0	0.0	0	0.0	0	0.0
7q	30	5.2	3.0	12.7	0.6	100.0	30	30	5.2	3.0	12.7	0	0.00	0	0.0	0.0	0.0	0	0.0	0	0.0
8p	23	3.4	2.5	9.2	0.7	100.0	23	23	3.4	2.5	9.2	0	0.00	0	0.0	0.0	0.0	0	0.0	0	0.0
8q	30	2.8	2.0	5.9	0.7	100.0	30	30	2.8	2.0	5.9	0	0.00	0	0.0	0.0	0.0	0	0.0	0	0.0
9p	30	4.4	2.2	8.8	0.5	100.0	30	30	4.4	2.2	8.8	0	0.00	0	0.0	0.0	0.0	0	0.0	0	0.0
10p	27	4.0	2.9	12.3	0.7	100.0	27	27	4.0	2.9	12.3	0	0.00	0	0.0	0.0	0.0	0	0.0	0	0.0
11p	30	3.2	2.1	7.6	0.6	100.0	30	30	3.2	2.1	7.6	0	0.00	0	0.0	0.0	0.0	0	0.0	0	0.0
11q	20	4.5	1.9	7.8	0.4	100.0	20	20	4.5	1.9	7.8	0	0.00	0	0.0	0.0	0.0	0	0.0	0	0.0
12p	30	2.4	1.3	5.3	0.5	100.0	30	30	2.4	1.3	5.3	0	0.00	0	0.0	0.0	0.0	0	0.0	0	0.0
12q	30	3.8	2.8	11.2	0.7	100.0	30	30	3.8	2.8	11.2	0	0.00	0	0.0	0.0	0.0	0	0.0	0	0.0
13q	30	5.1	3.1	12.2	0.6	100.0	30	30	5.1	3.1	12.2	0	0.00	0	0.0	0.0	0.0	0	0.0	0	0.0
14q	30	3.0	3.1	10.9	1.1	100.0	30	30	3.0	3.1	10.9	0	0.00	0	0.0	0.0	0.0	0	0.0	0	0.0
15q	28	3.8	2.3	8.8	0.6	100.0	28	28	3.8	2.3	8.8	0	0.00	0	0.0	0.0	0.0	0	0.0	0	0.0
17q	30	4.7	2.1	10.1	0.5	100.0	30	30	4.7	2.1	10.1	0	0.00	0	0.0	0.0	0.0	0	0.0	0	0.0
18p	28	3.9	2.4	8.0	0.6	100.0	28	28	3.9	2.4	8.0	0	0.00	0	0.0	0.0	0.0	0	0.0	0	0.0
18q	26	5.2	2.1	9.5	0.4	100.0	26	26	5.2	2.1	9.5	0	0.00	0	0.0	0.0	0.0	0	0.0	0	0.0
19p	30	3.0	2.1	10.0	0.7	100.0	30	30	3.0	2.1	10.0	0	0.00	0	0.0	0.0	0.0	0	0.0	0	0.0
20q	17	3.6	1.6	6.4	0.4	100.0	17	17	3.6	1.6	6.4	0	0.00	0	0.0	0.0	0.0	0	0.0	0	0.0
21q	30	3.6	1.9	7.9	0.5	100.0	30	30	3.6	1.9	7.9	0	0.00	0	0.0	0.0	0.0	0	0.0	0	0.0
