# Supplementary material for: Single-Molecule Telomere Assay via Optical Mapping (SMTA-OM) Can Potentially Define the ALT Positivity of Cancer
Source: Genes (Basel). 2023 Jun 16;14(6):1278. doi: 10.3390/genes14061278 (PMC10297966; doi:10.3390/genes14061278)
Supplement: Supplementary file 1 [file genes-14-01278-s001.zip › Supplementary Table S2_P values.pdf]

A	B	C	D
**P values between ALT+, IMR90-S, and TEL+ cell lines**			
	IMR90-S	UM-UC-3	LNCaP
**Overall telomere mean length**			
U2OS	0.073	0.594	0.881
SK-MEL-2	0.002	0.958	0.485
Saos2	0.661	0.180	0.732
**EndTel (-TFE) mean length**			
U2OS	0.072	0.003	0.040
SK-MEL-2	0.018	0.775	0.641
Saos2	0.489	0.028	0.106
**Super-long telomere length**			
U2OS	0.000	0.000	0.000
SK-MEL-2	0.007	0.004	0.000
Saos2	0.042	0.031	0.010
**Overall length coefficient of variation (CV)**			
U2OS	0.000	0.000	0.000
SK-MEL-2	0.000	0.000	0.000
Saos2	0.000	0.020	0.000
**P values within ALT+ cell lines**			
	U2OS	SK-MEL-2	Saos2
**ITS/Fusion %**			
U2OS	—	0.000	0.000
SK-MEL-2	0.000	—	0.374
Saos2	0.000	0.374	—
**ITS+/Fusion %**			
U2OS	—	0.005	0.748
SK-MEL-2	0.005	—	0.019
Saos2	0.748	0.019	—
**ITS-/Fusion %**			
U2OS	—	0.003	0.000
SK-MEL-2	0.003	—	0.239
Saos2	0.000	0.239	—
**Telomere-free end (TFE) %**			
U2OS	—	0.707	0.175
SK-MEL-2	0.707	—	0.242
Saos2	0.175	0.242	—
**ITS+/Fusion mean length**			
U2OS	—	0.370	0.998
SK-MEL-2	0.370	—	0.471
Saos2	0.998	0.471	—
**EndTel (-TFE) mean length**			
U2OS	—	0.009	0.229
SK-MEL-2	0.009	—	0.527
Saos2	0.229	0.527	—
